# Dissipation Dynamic and Final Residues of Oxadiargyl in Paddy Fields Using High-Performance Liquid Chromatography-Tandem Mass Spectrometry Coupled with Modified QuEChERS Method

**DOI:** 10.3390/ijerph15081680

**Published:** 2018-08-07

**Authors:** Xile Deng, Yong Zhou, Wenna Zheng, Lianyang Bai, Xiaomao Zhou

**Affiliations:** 1 Hunan Agricultural Biotechnology Research Institute, Hunan Academy of Agricultural Sciences, Changsha 410125, China; chemdxl@163.com (X.D.); qimiaobuchugan@126.com (Y.Z.); zhengwenna1994@163.com (W.Z.); bailianyang2005@aliyun.com (L.B.); 2 Long Ping Branch, Graduate School of Hunan University, Changsha 410125, China

**Keywords:** herbicide, degradation, residues, rice, HPLC/MS-MS

## Abstract

Oxadiargyl, which binds to the protoporphyrinogen oxidase IX to exhibit herbicide activity, is mainly used in the prevention of certain perennial broadleaved and grass weeds during the preemergence of rice in paddy fields. However, oxadiargyl affects the germination and seedling growth of rice, causing damage to the plant and reducing rice yield. Hence, monitoring fate and behaviour of oxadiargyl in rice paddy fields is of great significance. A modified quick, easy, cheap, effective, rugged, and safe (QuEChERS) sample preparation method coupled with high-performance liquid chromatography-tandem mass spectrometry (HPLC-MS/MS) was established in paddy water, paddy soil, rice straw, paddy hull, and brown rice. We validated this method for the first time in the analysis of the dissipation dynamic and residues of oxadiargyl over two years (2015–2016) at three sites in China. The average recoveries of oxadiargyl ranged from 76.0 to 98.8%, with relative standard deviations of 3.5–14.0%. The dissipation curves for paddy soil fit to a first-order kinetic equation, revealing that oxadiargyl degraded rapidly in paddy soil with half-lives (*t*_1/2_) of 4.5–7.6 days. The final oxadiargyl residues in all samples remained below the detection limit and the maximum residue limit in China (0.02 mg kg^−1^) and Japan (0.05 mg kg^−1^) during the harvesting dates and were not detected in rice straw.

## 1. Introduction

Paddy rice is mainly grown under submerged conditions in hot and humid areas (e.g., monsoonal Asia). Paddy rice is a major alimentary crop that feeds over half of the world population [1,2]. Although the market production of paddy rice has increased over the years, this growth is not sufficient to cover the rapid growth of the global population [3]. The amount produced could be improved by eliminating ‘harmful factors’ such as pests, fungi, and weeds [4]. In this regard, weeds can be conveniently and efficiently managed by herbicide application in replacement of other physical, mechanical, and cultural control methods. However, the use of herbicides has resulted in the exposure of crops to environmental contaminants, causing pollution and food safety problems [5,6].

**Figure 1:**
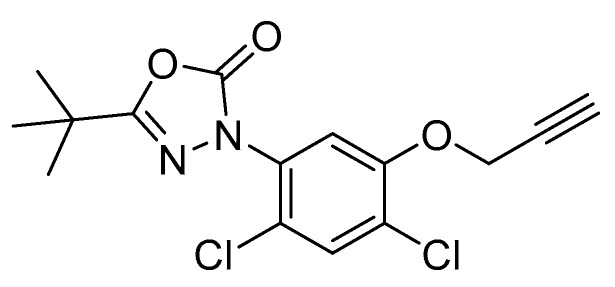
Chemical structure of oxadiargyl.

Oxadiargyl (3-[2,4-dichloro-5-(2-propynyloxy)phenyl]-5-(1,1-dimethylethyl)-1,3,4-oxadiazol-2 (3H)-one), with CAS (Chemical Abstracts Service) number of (39807-15-3), is an oxadiazolone class selective herbicide, and its chemical structure is depicted in Figure 1. The molecular weight of oxadiargyl is 341.1893. The octanol-water partition coefficient (log P) of it is 3.70 and the water solubility is 0.37 g L^−1^ at 20 °C. The herbicide activity of oxadiargyl lies in binding to the protoporphyrinogen oxidase IX as an inhibitor, preventing light-induced peroxidation and interrupting photosynthesis as a result [7,8]. This herbicide has been mainly used in the prevention of certain perennial broadleaved and grass weeds during the pre-emergence of rice [9,10] and the recommended rate of oxadiargyl in rice in China is 72–96 g a.i. ha^−1^ [11]. Although oxadiargyl protects rice crops from weeds damage, it also affects the germination and seedling growth of rice, causing damage to the plant and reducing rice yield [12,13,14]. For this reason, maximum residue limits (MRLs) have been established for oxadiargyl in rice in several countries (e.g., 0.01 mg kg^−1^ [15], 0.05 mg kg^−1^, and 0.02 mg kg^−1^ [16,17] in the European Union, Japan, and China, respectively). However, FAO (Food and Agriculture Organization of the United Nations), WHO (World Health Organization) and EPA (U.S. Environmental Protection Agency) have not set limits for this herbicide yet.

Considering that oxadiargyl is used in growing amounts and its residues affect rice growth, research on new detection methods of oxadiargyl residues in paddy field samples is quite significant. Oxadiargyl residues have been analyzed in environmental and food paddy field samples by gas chromatography−electron capture detector (GC−ECD) and GC coupled with mass spectrometry (GC−MS) methods [18,19,20]. Taking advantage of the high precision of LC, liquid chromatography methods have also been developed for determining oxadiargyl residues in paddy grain, straw, and soil samples by high-performance liquid chromatography (HPLC) and HPLC−electro−spray ionization−tandem mass spectrometry (HPLC−ESI−MS/MS) [21,22]. Despite the number of detection methods reported, only two studies have studied the dissipation dynamic of oxadiargyl, and studies on the final residues of oxadiargyl have been reported. These two previous articles were mainly focused on the metabolism of oxadiargyl in water samples under laboratory conditions or on studying the association interactions between soil particles and oxadiargyl in calcareous fields [8,23]. The fate and behaviour of oxadiargyl in paddy fields have not been adequately monitored and further studies are required.

Recently, four oxadiargyl extraction methods from rice samples have been described: (i) liquid–liquid extraction (LLE) [18]; (ii) quick, easy, cheap, effective, rugged, and safe (QuEChERS) [20,24,25]; (iii) solid-phase micro-extraction (SPME) [19]; and (iv) magnetic micro−solid phase extraction (M−*µ*−SPE) methods [26]. The LLE procedure to extract oxadiargyl consisted of immersion in dichloromethane, extraction in the separating funnel, and purification using the column filled with activated carbon and Florisil [18]. However, this method is limited as it requires large amounts of toxic organic solvents, takes a relative long period of time, is not sensitive enough, and so on. Compared with this traditional method, QuEChERS is preferably used for oxadiargyl residue analysis because of its unique convenience and suitability for avoiding superfluous analytical procedures using low maintenance and cheap instruments [27,28]. In addition, the QuEChERS method could be applied to rapidly detect oxadiargyl residues in different samples rapidly by modifying the chromatography device with various detectors [29,30,31,32].

This study was aimed to develop a modified QuEChERS sample preparation method coupled with HPLC−MS/MS. This method was used for the analysis of the dissipation dynamics and final remaining residues of oxadiargyl at harvest date in paddy water, paddy soil, rice straw, paddy hull, and brown rice matrices. The experiment results obtained with this method can help determining the fate and behaviour of oxadiargyl in paddy fields and assess its environmental risk. In addition, it can also provide a valuable guide for the proper and safe use of oxadiargyl under field trials.

## 2. Materials and Methods

### 2.1. Chemicals and Materials

An analytical standard of oxadiargyl (99.0%) was purchased from Dr. Ehrenstorfer GmbH (Augsburg, Germany). HPLC grade acetonitrile, methanol, and water were obtained from Merck KGaA (Darmstadt, Germany). All the solvents used in this study were further purified by redistillation. Analytical reagent grade sodium chloride (98.0%), and anhydrous magnesium sulphate (97.0%) were purchased from Sinopharm Chemical Reagent Co., Ltd. (Shanghai, China). Primary secondary amine (PSA) sorbent was obtained from CNW Technologies GmbH (Duesseldorf, Germany). A commercial 80% WDG (water dispersible granule) formulation (80% oxadiargyl) was purchased from Hefei Xingyu Chemical Co., Ltd. (Hefei, China).

A stock solution of oxadiargyl (100 mg L^−1^) was prepared in methanol and stored in a freezer at −4 °C. The work stock solutions (5, 10, 50, 100, 500, and 1000 μg L^−1^) were prepared by diluting the stock solution with methanol. All the stock solutions were stored at −4 °C until the time of HPLC−MS/MS analysis.

The linearity of the method in the analysis of paddy water, paddy soil, rice straw, paddy hull, and brown rice samples was studied by building matrix matched standard calibration curves over a 5–1000 µg L^−1^ range with six calibration points (5, 10, 50, 100, 500, and 1000 μg L^−1^), with five replicates being carried out for each concentration. Least squares regression of the peak area to concentration plots of the oxadiargyl standard was used to confirm the linear calibration curves. The matrix effects (ME) were calculated as the ratio between the calibration curve slopes of the matrix matched standards and the solvent based standards. The standard curves, constructed by plotting the peak areas against the analyte concentrations, were used for quantification. The calibration curves for the different samples are described in Table 1.

### 2.2. Field Experiments

Field experiments were carried out to assess the final residues and dissipation dynamics. These experiments were conducted in 2015−2016 at three locations: Heilongjiang Province (45°51′45.53″ N, 126°15′27.82″ E), Anhui Province (33°59′36.08″ N, 116°49′44.38″ E), and Hunan Province (28°27′49.84′′ N, 113°19′47.37″ E). The treatment fields used for this study consisted of plots with an area of 30 m^2^ with three replicates. Irrigation ditches with widths of 1 m separated the plots.

To study the dissipation dynamics of paddy water, paddy soil, rice straw, paddy hull, and brown rice, commercial oxadiargyl formulation was dissolved in water, mixed with soil, and sprayed with the sand mix broadcast on each experiment plot in paddy fields, with three replicates. The dosage used in this study was 126 g a.i ha^−1^. The field experiments were performed within 3–7 days before transplanting rice. All samples were randomly collected at 0, 1, 3, 5, 7, 14, 21, 28, and 45 days at harvest time and stored in a freezer at −4 °C until the time of analysis.

To investigate the final residues of oxadiargyl in harvested paddy water, paddy soil, rice straw, paddy hull, and brown rice samples, a number of experiments were performed at two dosage levels (84 and 126 g a.i ha^−1^). These experiments were repeated three times. Paddy water, paddy soil, rice straw, paddy hull, and brown rice samples were randomly collected from each plot. Then, the samples were stored in a freezer at −4 °C and subsequently analysed to obtain the final residue data.

### 2.3. Sample Preparation

Harvested rice straw, paddy hull, and brown rice samples were mixed with a grinder and the solid samples were dried. All samples were placed in polyethylene bags and stored in a refrigerator at −4 °C before the time of analysis.

Rice straw, paddy hull, and brown rice samples (10.0 g) were added to 100 mL centrifuge tubes, and 10 mL of pure water and 40 mL of acetonitrile were subsequently added. After the tubes were vortexed for 2 min, 5.0 g of NaCl were added to each tube. Then, the samples were vortexed for 3 min and centrifuged at 4000 rpm for 3 min.

In our study, a modified QuEChERS sample preparation method was developed to analyze all the samples (paddy water, paddy soil, rice straw, paddy hull, and brown rice). Then, 1 mL of the extract was transferred to a 2 mL centrifuge tube containing 100 mg of PSA, and the tube was vortexed for 1 min. Then, each tube was centrifuged at 15,000 rpm for 3 min, and the extract was filtered through a 0.22 µm nylon filter to an autosampler vial for HPLC−MS/MS analysis.

Fortified recovery experiments were carried out by spiking the standard solutions into blank samples. After standing for 30 min at room temperature, these samples were extracted by the sample preparation method as described above. The fortification recoveries and reproducibility experiments were carried out for each sample in five replicates at three fortification levels (20, 200, and 2000 μg kg^−1^). Considering the dilution carried out during sample preparation, the actual concentrations for HPLC−MS/MS analysis were 5, 50, and 500 μg L^−1^.

### 2.4. Instrumentation

A Thermo UltiMate 3000 HPLC system and a Thermo TSQ Endura tandem mass spectrometer were used to detect oxadiargyl in the samples. A Hypersil GOLD reverse phase C18 HPLC column (100 mm × 2.1 mm × 1.9 µm) containing ultra high purity ZORBAX RX−SIL porous silica was used to separate the sample components (35 °C). Methanol and water containing 0.1% (*v*/*v*) formic acid were used as the mobile phase with a flow rate of 0.3 mL min^−1^. The mobile phase gradient program was as follows: 20% methanol for 0.5 min, increased to 95% methanol from 0.5 min to 2.0 min, maintained at 95% methanol for 2.5 min, decreased to 20% methanol, and maintained for 0.1 min. The nebulizing gas pressure was 40 psi, while the injection volume was set to 10 µL.

The spectra were acquired via positive electrospray ionization mode for all oxadiargyl analyses on a Thermo TSQ Endura vector. The parameters used for oxadiargyl analyses are described in Table 2.

### 2.5. Statistical Analysis

Microsoft Excel software was used to analyse the oxadiargyl dissipation curves via nonlinear regression. First order kinetics (C = C_0_e^−kt^) were used to study the oxadiargyl residue dissipation process, where C is the concentration (μg kg^−1^) at time t (days); C_0_ is the initial concentration (μg kg^−1^); and k is the first order rate constant (day^−1^), which is independent of C and C_0_. The correlation coefficient (R^2^) is the representation of the congruence between the first order kinetic model and the data. The half life (*t*_1/2_; time required for the disappearance of 50% of the oxadiargyl concentration) was calculated using the Hoskins formula: *t*_1/2_ = ln 2/k.

## 3. Results and Discussion

### 3.1. Method Validation

To validate the efficiency of the method, working solutions of oxadiargyl were prepared at 20, 200, and 200 μg kg^−1^ for the paddy water, paddy soil, rice straw, paddy hull, and brown rice samples. All quantitative analyses were conducted using matrix matched calibration curves, with five replicates being analyzed for each matrix. The fortified recoveries and relative standard deviations (RSDs) obtained are listed in Table 3. The average recoveries of oxadiargyl for the five matrices (paddy water, paddy soil, rice straw, paddy hull, and brown rice) ranged from 76.0 to 98.8% with RSDs of 3.5–14.0%. These results revealed that the proposed method possessed good accuracy and repeatability for oxadiargyl residue analysis according to the standard of pesticide residue analysis established by the European Commission [33]. According to Chen et al., the recoveries of oxadiargyl ranged from 88 to 96%, and RSD values were 5.6–13.3% in rice samples [18]; according to Shi et al., the recoveries of oxadiargyl in rice samples varied from 82.9 to 84.8%, and RSD values were 0.2–2.7% [20]; according to Es-haghi et al., the recoveries of oxadiargyl in rice samples were 73–79% and RSD values were less than 10% [26]; according to Su et al., the recoveries of oxadiargyl were 83.4–93.4%, and RSD values were 10.2–11.6% in rice samples [30]. In our study, the recoveries of oxadiargyl in rice samples varied from 76.0 to 96.5%, which had no significant difference than those reported by Chen et al. and Su et al., but were higher than those reported by Es-haghi et al. RSD values ranged from 4.0 to 14.0%, which had no significant difference than those reported by Chen et al., but were higher than those reported by Shi et al.

The limits of quantification (LOQs) in paddy water, paddy soil, rice straw, paddy hull, and brown rice samples were 20 μg kg^−1^ at a signal to noise (S/N) ratio of 10. The limits of detections (LODs) in paddy water, paddy soil, rice straw, paddy hull, and brown rice samples were 6 μg kg^−1^ at an S/N ratio of 3. 

### 3.2. Dissipation of Oxadiargyl in Field Trials

**Figure 2:**
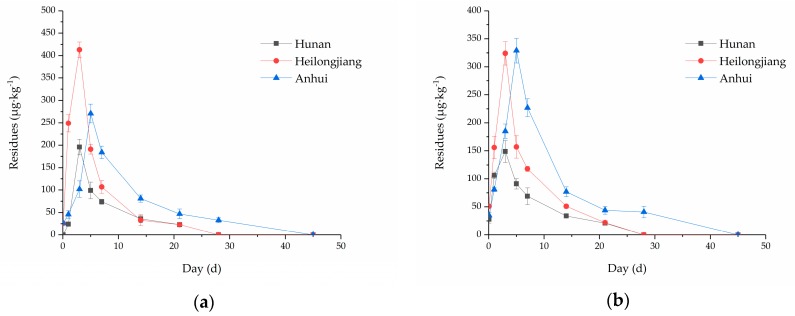
Dissipation curves of oxadiargyl in paddy soil for three locations (Hunan, Heilongjiang and Anhui Province) over two years described by a first order kinetic model: (**a**) 2015 and (**b**) 2016.

The dissipation curves of oxadiargyl for the paddy soil sample from the Hunan, Heilongjiang, and Anhui Provinces in 2015 and 2016 are shown in Figure 2, while the dissipation data for oxadiargyl and the pH of the paddy soil are summarized in Table 4. In this study, the dissipation curves of the paddy soil sample fit well to a first order kinetic equation. The residuals of oxadiargyl increased quickly right after using the herbicide, reaching maximum values of 149–413 μg kg^−1^ for all locations after five days, before decreasing fast to a low steady concentration after 25–40 days with *t*_1/2_ values ranging from 4.5 to 7.6 days. These results revealed that oxadiargyl degraded fast, given its non persistent immobile nature. In Mahmoudi et al., the *t*_1/2_ values were calculated to be about 14.5 days in Dashtnaz and about 25.5 days in Gharakhil soils. It is worth mentioning that the *t*_1/2_ values in our research obtained herein were significantly lower than those reported in the above literature (14.4–25.5 days) for soil samples [24]. The different dissipation rates obtained herein could be explained by the monsoon climate of China, which has a relative long raining season as compared with Iran. Thus, rainwater varies the solid to solution ratio and may weaken the association interactions between the soil particles and oxadiargyl. Thus, oxadiargyl was dissipated more rapidly in our study because these interactions protect this compound from degradation.

As shown in Table 4, the samples from the Heilongjiang province exhibited slightly faster dissipation rates (*t*_1/2_ of 4.5–5.0 days) than the Hunan and Anhui samples (*t*_1/2_ of 6.4–6.7 and 7.4–7.6 days, respectively). Considering that the hydrolysis of heterocyclic oxadiazoline is the main mechanism for degradation of oxadiargyl, pH was reported to be a critical factor affecting the dissipation of oxadiargyl. Thus, oxadiargyl was more unstable under alkaline conditions because the *t*_1/2_ in alkaline media was significantly lower than those obtained under acidic and neutral conditions [8]. Hence, the rapid degradation rate for the Heilongjiang samples could be produced by the relatively higher pH of the soil, which can enhance the rate of alkaline hydrolysis.

Herbicidal ionic liquids (HILs), containing herbicidal anions with the counter cations, were introduced by Pernak et al. in 2011 for the first time [34]. As a result of their novel and unique physical−chemical properties and excellent biological activity, HILs were defined as the third generation of ionic liquids (ILs) [35]. HILs were reported to be improving the herbicidal efficacy compared with commercial herbicide and significantly reducing the herbicide usage in field trials. To date, there are numerous reports that describe the HILs of commercial herbicide, for example, 2,4-D, glyphosate, fomesafen, bentazone, metsulfuron−methyl, picloram, MCPP (2-(4-chloro-2-methylphenoxy)propanoic acid), MCPA (4-chloro-2-methylphenoxyacetic acid), MCPB (4-(4-chloro-2-methylphenoxy)butanoic acid) [36,37,38,39,40,41,42,43,44]. Hence, the application of oxadiargyl in the form of HILs could be an effective choice to enhance its herbicide activity and reduce the consumption.

### 3.3. Final Residues of Oxadiargyl in Field Trials

The final residues of oxadiargyl at harvest date in the paddy water, paddy soil, rice straw, paddy hull, and brown rice samples collected during the field trials are summarized in Table 5. As shown in Table 5, the oxadiargyl residues were very low in all matrices. The residual oxadiargyl concentrations in paddy water, paddy soil, rice straw, paddy hull, and brown rice all remained below the LOQ (20 μg kg^−1^). The MRL established in China and in Japan for oxadiargyl in brown rice is 0.02 mg kg^−1^ and 0.05 mg kg^−1^, respectively. Therefore, when oxadiargyl was used under the designed conditions, its residual concentration remained below the MRL in China and Japan, indicating that oxadiargyl can be safely applied to paddy rice at a dosage of 126 g a.i ha^−1^. We believe that this work can serve as a valuable guide for the proper and safe use of oxadiargyl in paddy rice fields.

## 4. Conclusions

In summary, a modified QuEChERS sample preparation method coupled with HPLC−MS/MS was developed and validated for the analysis of oxadiargyl residues in paddy fields. By this method, the dissipation dynamic and final residues of oxadiargyl were studied in an attempt to obtain the *t*_1/2_ and final residue data. The dissipation curves for paddy soil fit to a first order kinetics equation, indicating that oxadiargyl degraded rapidly in paddy soil with *t*_1/2_ values of 4.5−7.6 days. Oxadiargyl residues were undetected for rice straw, and were below 20 μg kg^−1^ for paddy water, paddy soil, paddy hull and brown rice samples on the harvest day. The concentration of oxadiargyl residues remained below the LOQ and MRL values established for China.

## Figures and Tables

**Table 1:** Linear equations, correlation coefficients (R^2^) and matrix effect (ME) determined for paddy water, paddy soil, rice straw, paddy hull, and brown rice.

Matrix	Linear Equation	Correlation Coefficient	ME
Paddy water	y = 913.90x + 1757.7	0.9999	1.0430
Paddy soil	y = 837.03x + 3184.3	0.9992	0.9550
Rice straw	y = 727.37x + 2234.9	0.9979	0.8300
Paddy hull	y = 755.67x − 5933.9	0.9987	0.8623
Brown rice	y = 834.12x + 1753.2	0.9997	0.9518

**Table 2:** High-performance liquid chromatography (HPL—Ctandem mass spectrometry (MS/MS) detection parameters for oxadiargyl.

Compound	*t_R_* (min)	Precursor Ion (*m*/*z*)	Product Ion (*m*/*z*)	RF Lens (V)	Collison Energy (V)
Oxadiargyl	4.10	341.07	222.89	139	16
	4.10	341.07	150.93	139	26

**Table 3:** Fortified recoveries (*n* = 5) and relative standard deviations (RSDs) of oxadiargyl in paddy water, paddy soil, rice straw, paddy hull, and brown rice. LOQ—limits of quantification; LOD—limits of detections.

Matrix	Fortified Level (μg kg^−1^)	Average Recovery (%)	RSD (%)	LOQ	LOD
				(μg kg^−1^)	(μg kg^−1^)
Paddy water	20	80.4	4.3	20	6
	200	90.1	3.5		
	2000	98.8	3.8		
Paddy soil	20	96.5	6.1	20	6
	200	88.2	7.6		
	2000	95.9	4.0		
Rice straw	20	84.2	14.0	20	6
	200	91.2	11.2		
	2000	89.6	8.5		
Paddy hull	20	76.0	6.5	20	6
	200	86.1	6.0		
	2000	77.1	6.7		
Brown rice	20	85.5	8.7	20	6
	200	92.8	5.8		
	2000	93.8	7.1		

**Table 4:** Dissipation, *t*_1/2_ and correlation coefficients of oxadiargyl in paddy soil.

Location	Year	Kinetic Equation ^1^	R^2^	*t*_1/2_ (days)	pH Value
Hunan	2015	C = 0.1945e^−0.109t^	0.9226	6.4	6.8
	2016	C = 0.1632e^−0.103t^	0.9588	6.7	
Heilongjiang	2015	C = 0.4284e^−0.154t^	0.9067	4.5	8.2
	2016	C = 0.3668e^−0.138t^	0.9662	5.0	
Anhui	2015	C = 0.3553e^−0.091t^	0.9627	7.6	5.9
	2016	C = 0.4117e^−0.094t^	0.8983	7.4	

^1^ The data were fitted to a first order kinetic equation.

**Table 5:** Final residues of oxadiargyl in paddy water, paddy soil, rice straw, paddy hull and brown rice on the harvest date.

Matrix	Year	Dosage (g a.i. ha^−1^)	Residue (μg kg^−^^1^)		
			Hunan	Heilongjiang	Anhui
Paddy water	2015	84	<LOQ	<LOQ	<LOQ
	2016	126	<LOQ	<LOQ	<LOQ
Paddy soil	2015	84	<LOQ	<LOQ	<LOQ
	2016	126	<LOQ	<LOQ	<LOQ
Rice straw	2015	84	<LOQ	<LOQ	<LOQ
	2016	126	<LOQ	<LOQ	<LOQ
Paddy hull	2015	84	<LOQ	<LOQ	<LOQ
	2016	126	<LOQ	<LOQ	<LOQ
Brown rice	2015	84	<LOQ	<LOQ	<LOQ
	2016	126	<LOQ	<LOQ	<LOQ
